# Quantitative T2 Magnetic Resonance Imaging Compared to Morphological Grading of the Early Cervical Intervertebral Disc Degeneration: An Evaluation Approach in Asymptomatic Young Adults

**DOI:** 10.1371/journal.pone.0087856

**Published:** 2014-02-03

**Authors:** Chun Chen, Minghua Huang, Zhihua Han, Lixin Shao, Yan Xie, Jianhong Wu, Yan Zhang, Hongkui Xin, Aijun Ren, Yong Guo, Deli Wang, Qing He, Dike Ruan

**Affiliations:** 1 The Third School of Clinical Medicine, Southern Medical University, Guangzhou, Guangdong, China; 2 Department of Orthopedic Surgery, Navy General Hospital, Beijing, China; 3 Department of Radiology, Navy General Hospital, Beijing, China; Vanderbilt University, United States of America

## Abstract

**Objective:**

The objective of this study was to evaluate the efficacy of quantitative T2 magnetic resonance imaging (MRI) for quantifying early cervical intervertebral disc (IVD) degeneration in asymptomatic young adults by correlating the T2 value with Pfirrmann grade, sex, and anatomic level.

**Methods:**

Seventy asymptomatic young subjects (34 men and 36 women; mean age, 22.80±2.11 yr; range, 18–25 years) underwent 3.0-T MRI to obtain morphological data (one T1-fast spin echo (FSE) and three-plane T2-FSE, used to assign a Pfirrmann grade (I–V)) and for T2 mapping (multi-echo spin echo). T2 values in the nucleus pulposus (NP, n = 350) and anulus fibrosus (AF, n = 700) were obtained. Differences in T2 values between sexes and anatomic level were evaluated, and linear correlation analysis of T2 values *versus* degenerative grade was conducted.

**Findings:**

Cervical IVDs of healthy young adults were commonly determined to be at Pfirrmann grades I and II. T2 values of NPs were significantly higher than those of AF at all anatomic levels (P<0.000). The NP, anterior AF and posterior AF values did not differ significantly between genders at the same anatomic level (P>0.05). T2 values decreased linearly with degenerative grade. Linear correlation analysis revealed a strong negative association between the Pfirrmann grade and the T2 values of the NP (P = 0.000) but not the T2 values of the AF (P = 0.854). However, non-degenerated discs (Pfirrmann grades I and II) showed a wide range of T2 relaxation time. T2 values according to disc degeneration level classification were as follows: grade I (>62.03 ms), grade II (54.60–62.03 ms), grade III (<54.60 ms).

**Conclusions:**

T2 quantitation provides a more sensitive and robust approach for detecting and characterizing the early stage of cervical IVD degeneration and to create a reliable quantitative in healthy young adults.

## Introduction

The spine is exposed to a more complex set of compressive and torsional forces than any other skeletal array. Neck or upper back pain in humans is common [1]–[3], mainly due to intervertebral disc (IVD) degeneration [3]. Although children and adolescents rarely experience persistent or recurrent neck stiffness or pain, a number of modern psychological factors, such as environment and emotion, have been linked with a younger onset of disc degeneration [2]. Several researchers reported that at least one type of degenerative magnetic resonance imaging (MRI) finding was present in both the lumbar and cervical spine in 78.7% of subjects, which suggests that disc degeneration can occur concurrently in asymptomatic subjects [4], [5]. Despite the high prevalence of IVD degeneration and chronic neck pain throughout the world [3], diagnosis of degeneration in the early stages of symptomatic disease is elusive in clinical practice [6].

Morphological MRI is a well-established method for the evaluation of IVDs, and it allows disc degeneration to be graded using the widely accepted Pfirrmann grade [7]. Although the Pfirrmann grading system provides a semi-quantitative evaluation of disc degeneration, it does not provide reliable quantification of the degenerative grade in the early stages of degeneration, which are characterized by a loss of water or proteoglycan (PG) in an intact disc. Therefore, a more sensitive technique is needed to quantify the biochemical changes that occur during the early stages of IVD degeneration. Such a method would allow more accurate evaluation of new therapies for the treatment of IVD degeneration, such as injection of recombinant growth factor, gene therapy, stem cell therapy, and tissue engineering strategies [8]–[10].

The T2 value is defined as the transverse magnetization relaxation of maximum signal intensity (SI) attenuation up to 37%. The T2 relaxation time has been reported to reflect the environment of molecules produced in the IVDs, which include protein, neutral fat, collagen, and other solutes[11]. Several studies have described the benefits of T2 mapping as a technique for biochemical and structural analysis of cartilage tissue of the spine, IVD and other joints [12]–[14]. Good correlation between T2 mapping values and water or PG content in lumbar IVD tissue has widely validated [15]–[19]. Increased disc water or glycosaminoglycan content, the primary component of PG, is associated with increased T2, whereas increased collagen content contributes to decreased T2 [16]. Previous studies demonstrated that T2 relaxation time was directly correlated to water and PG content of human lumbar IVDs *ex vivo* [16] and correlated significantly with disc degeneration *in vivo* [17], [18] as well as clinical symptoms. Watanabe et al.[20] reported that subjects older than 20s old showed degeneration in the lumbar IVD according to axial T2 mapping, and this result was confirmed by histologic data [11], [21]. Results of previous studies also suggested that different tissue compartments within the IVD (i.e., the annulus fibrosus (AF) and nucleus pulposus (NP)) could be characterized based on T2 values [17], [22].

IVD degeneration is known to be common among asymptomatic young adults, and few studies on young population cohort demonstrate that degenerative MRI findings are also present in the cervical spine among young adults [23]. Therefore, it is well worth investigating the early stage of biochemical components of cervical IVDs in young healthy adolescents. To the best of our knowledge, no previous studies have been conducted to evaluate early degenerative changes in the cervical spine of young adults using functional MRI techniques that are sensitive to such changes. The purpose of this study was to evaluate IVDs in the cervical spine using T2 mapping values and T2-weighted MRI in a population of asymptomatic young adults and to compare the usefulness of T2 mapping values as indicators of IVD degeneration with the conventional indicator (i.e., Pfirrmann degenerative grade), sex, anatomic site, and disc level were also considered in the analysis.

## Materials and Methods

### Ethics Statement

The study was approved by the institutional review board of the Navy General Hospital and all participants provided written informed consent prior to enrollment.

### Study Sample

The study population consisted of 73 volunteers (35 men and 38 women; mean age, 22.80±2.11 yr; range, 18–25 years). All patients underwent standard clinical and T2 mapping MRI of the cervical spine. Inclusion criteria consisted of age younger than 25 years, no history of chronic neck pain, no prior back surgery, and without trauma history of the spine, metabolic disease, autoimmune disease, and osteoarticular disease. Subjects were excluded if they failed to meet the above criteria or if they had any indicative suggestive of neurological symptoms, including neck or arm weakness, numbness, or tingling. Three participants were excluded from analysis due to chronic neck pain and osteoarticular disease. In total, 350 cervical discs (70 of 73 subjects) were examined. The study evaluated discs segments between C2 and C7 for a total of discs.

### MRI image acquisition

MRI was performed using a 3.0-T GE Signa Echo-Speed MR scanner (GE Medical Systems, Milwaukee, WI, USA). All MRI images in this study were obtained in the afternoon to minimize the diurnal variation of T2 values in the IVDs [24].

Sagittal T1-weighted fast spin echo (FSE) and sagittal, transversal, and axial T2-weighted FSE sequences were used for morphological MRI (for detailed sequence parameters see Table 1). The sagittal T2 weighted images (WIs) were used for visual Pfirrmann grading of IVD degeneration. Next, a T2 map was created using the T2 values in the midsagittal section from sagittal sections centered on the cervical midline region with optimized 8 echo multi-spin echo (Repetition time /first,last time echo time, TR/(fTE, lTE), 1500/8.5, 17.0, 25.5, 34, 42.4, 50.9, 59.4, 67.9, field of view (FOV)  = 20 mm×20 mm, section thickness  = 3.0 mm, matrix  = 256×160, number of signal intensity acquisitions  = 1, and total examination time 4 min and 27 s) obtained using an ADW 4.3 workstation (Functool, GE Medical Systems, Milwaukee WI, USA). However, the first echo from the multi-spin system was excluded to minimize the effect of the stimulated echo [14]. A single midline sagittal section was positioned parallel to each cervical IVD from C2–3 to C6–7. The T2 maps were computed in each pixel from the SI in the respective TE using the following formula: *SI  = e^−TE/T2^*.

**Table 1:** Imaging parameters.

Sequence	T1WI-FSE sag	T2WI-FSE cor	T2WI-FSE tra	T2WI-FSE sag	T2 map sag
Repetition (milliseconds, ms)	500	2560	3000	2300	1500
Echo time (milliseconds, ms)	11.5	102	103.0	108.7	8.5–67.9
Field of view (millimeter, mm)	24×24	24×24	16×16	24×24	20×20
Matrix	320×224	320×224	320×224	320×192	256×160
Slice thickness (millimeter, mm)	3	3	3	3	3
Interslice gap (millimeter, mm)	0.5	0.5	0.5	0.5	0.6
Number of slices	10	10	10	10	64
Echo trains/slice	4	18	19	18	—
Band width(Kilo hertz, KHz)	62.50	31.25	31.25	31.25	31.25
Number of signal-intensity acquision	2	4	4	6	1
Examination time	01:02	02:18	02:30	03:06	04:27

MR parameters for morphological and quantitative imaging. FSE = fast spin echo; sag = sagittal; tra = transversal; cor = coronal; WI = weighted image.

### Image analysis

**Figure 1:**
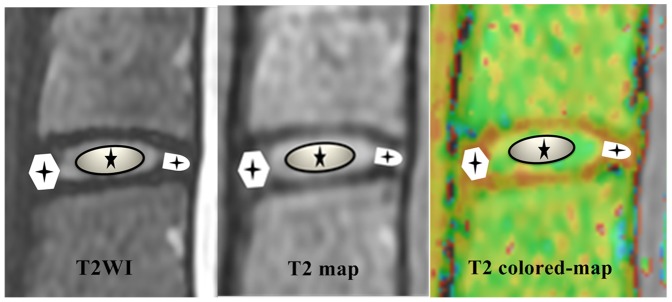
For the representative intervertebral disc, regions of interest (ROIs) evaluation on the sagittal T2WI, an ellipse ROI was selected for the nucleus pulposus (NP). Suitable ROIs for both the anterior and posterior annulus fibrosus (AF) were selected. Then these ROIs in T2WI were copied to the T2 map and T2 colored-map. T2 values were measured.

All Pfirrmann grade evaluations were performed by two radiologists in consensus. One radiologist had more than 10 years of experience and a special interest in musculoskeletal radiology [observer A], and the other had more than 20 years of experience in orthopedic radiology [observer B]. On the sagittal T2-weighted FSE images, the five IVDs (C2–7) of the cervical spine were analyzed. There is a gradual transition from AF to NP tissue and an irregular area of cervical sagittal image often is present, so it is sometimes challenging to define a clear border between the two tissues. Therefore, to evaluate regions of interest (ROIs) in a standardized and reproducible way, we decided to use the method described by Yi [25] and Nagashima [26]. With T2-WIs as the reference, a radiology trainee (L.X.S) with 9 years of experience reading MRI images manually draw ROIs over the T2 map of the discs. ROIs included the NP, anterior AF, and posterior AF (Fig. 1). The shape of the ROIs in the NP of the map was determined such that the quadrangle formed by the anterior and posterior edges of the upper and lower end plates in contact with the IVD to be measured was defined as the intervertebral area. A shape similar to the intervertebral area but with one-quarter the area was drawn in cervical IVDs. The geometrical center of the shape was matched to the center of intensity, so an ellipse was drawn. Two appropriate ROIs were delineated for anterior AF and posterior AF according to their irregular shapes. When an apparent tear was noted in the annulus, the abnormal signal areas were excluded in the ROIs. All ROIs were selected on the morphological images and transferred via “copy and paste” onto the T2 maps with areas of 13.6±2.9 mm^2^ and 5.1±1.2 mm^2^ to cover the NP and anterior AF or posterior AF respectively; these values were calculated from 1050 representative ROIs. T2 values are given as Mean ± SD.

### Inter- and intraobserver analysis

An interobserver evaluation of T2 WIs generated for the 1050 representative regions from 350 discs by two independent observers with different skill levels was conducted. Both observers had at least 10 years' experience in musculoskeletal MRI evaluation. In addition, both observers performed the same analysis twice, with a month in between analyses, to assess intraobserver agreement.

### Statistical analysis

Statistical analyses were conducted and graphs were generated using SPSS 19.0 (SPSS Inc., Chicago, IL, USA). We compared median T2 mapping values between men and women for both NP and AF according to the anatomic level of each disc using one-way analysis of variance (ANOVA). To evaluate the reliability of Pfirrmann grading, we tested intraobserver and interobserver agreement using the kappa statistic. The degree of internal consistency was assessed as follows: *k*≥0.7, strong consistency; 0.7>*k*≥0.4, moderate consistency; and *k*<0.4, weak consistency. Univariate ANOVA and post-hoc tests were used for Pfirrmann group comparisons. Welch's correction was used when heteroscedasticity was detected. In addition, Spearman rank correlation analysis was performed to assess the correlation between T2 values of NP or AF and Pfirrmann grading. Box plots and receiver operating characteristic (ROC) were generated. The level of statistical significance (alpha) was set at 0.05.

## Results

### Morphological MRI findings

Table 2 shows the characteristics of all analyzed discs sorted based on Pfirrmann degeneration grade: 79 discs (22.57%) were classified as grade I, 145 discs (41.42%) as grade II, 123 discs (35.14%) as grade III, and 3 disc (0.85%) had a collapsed disc space (grade IV). No discs were graded as Pfirrmann V in this study.

**Table 2:** Degeneration grade according to Pfirrmann scale, sex, and disc level of the cervical intervertebral discs of healthy young adults analyzed in study.

Degeneration	Sex		Disc Levels					
Grade	Male	Female	C2-3	C3-4	C4-5	C5-6	C6-7	Total
I	42	37	15	15	15	13	21	79
II	75	70	33	31	30	23	28	145
III	51	72	22	24	25	32	20	123
IV	2	1	0	0	0	2	1	3
Total	170	180	70	70	70	70	70	350

### Inter- and Intraobserver agreement

The Pfirrmann grading reproducibility of the two readers was high. The intraobserver test yielded *k* values ranging from 0.741 (P = 0.000) to 0.755 (P = 0.000), whereas the interobserver test produced *k* values of 0.851 (P = 0.000) (Table 3). The main reason for disagreement in grading discs was the difficulty in distinguishing the border between the AF and the NP.

**Table 3:** Intraobserver and interobserver reliability of the Pfirrmann grade.

Observer	*k* value	P value
intraobserver		
A1-A2	0.755	0.000
B1-B2	0.741	0.000
interobserver		
A1-B1	0.815	0.000

### Anatomical level and gender

**Figure 2:**
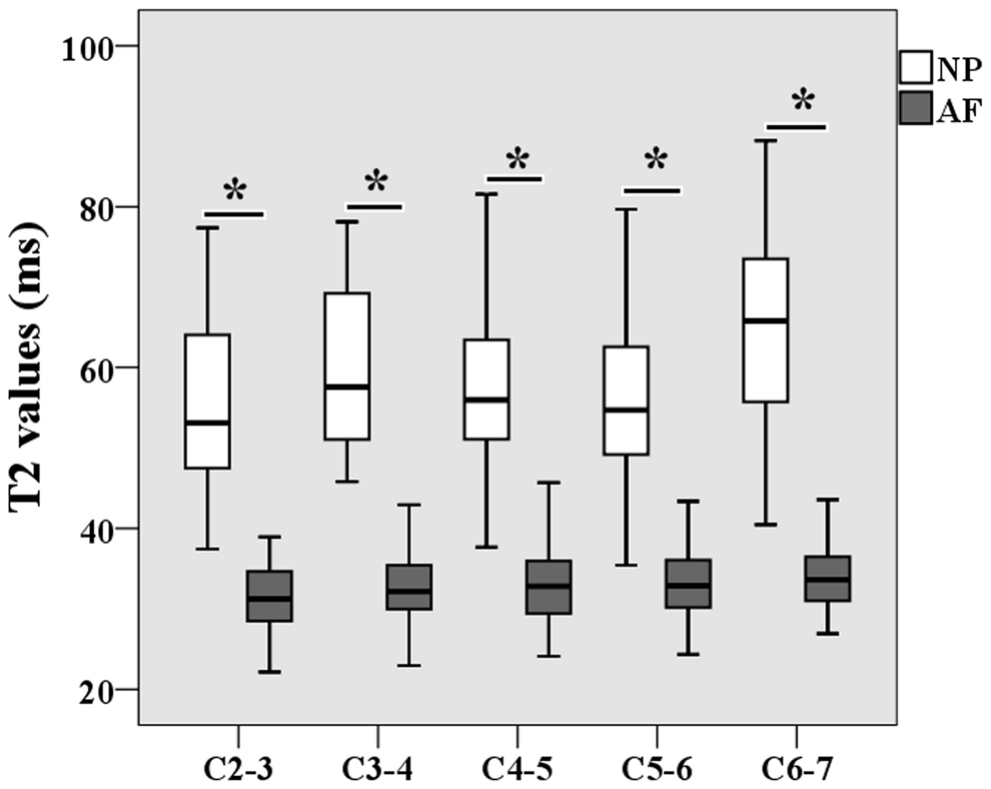
Box plots of T2 value of both nucleus pulposus (NP) and annulus fibrosus (AF) according to the anatomic level of each disc. The boxes represent the median and the interquartile range, with the vertical lines showing the range. *P<0.000.

**Figure 3:**
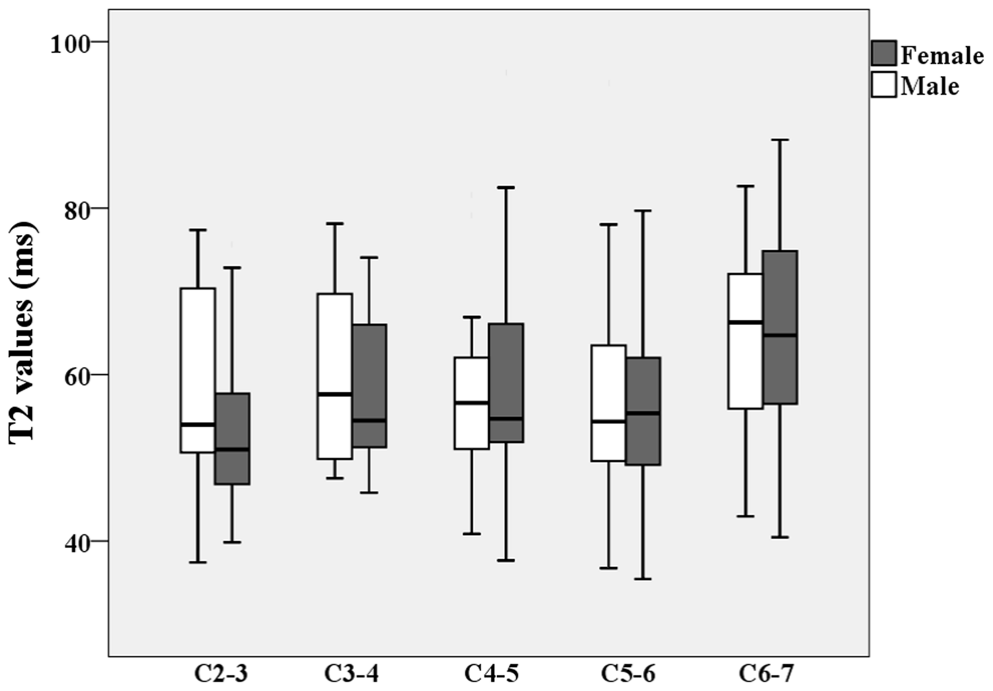
Box plots of T2 value of nucleus pulposus (NP) by sex according to the anatomic level of each disc. White: Male; Black: Female. The boxes represent the median and the interquartile range, with the vertical lines showing the range.

Table 4 lists the T2 values for both NP and AF at the different anatomic level of each disc for each gender in the study population. For both sexes, the T2 values of the NP were significantly higher than the AF values at all anatomic levels (P<0.000). T2 values of the AF slightly increased with caudal distance down the spinal column, and the maximum T2 values for both the NP and AF were in the C6–7 discs (Fig. 2). No significant differences in T2 values at different anatomic levels were found between the sexes. Figure 3 shows the box plot for NP T2 values at different cervical levels for male and female subjects. T2 values of the NP tended to decrease from C2–3 to C4–5 and then increase from C4–5 to C6–7. The T2 values of the AF tended to increase from C2–3 to C6–7.

**Table 4:** T2 values of male and female on nucleus pulposus and annulus fibrosus according to the anatomic level of each disc expressed as median and interquartile range.

	T2 values (ms) of the NP			T2 values (ms) of the AF		
Disc Levels	Male	Female	P value	Male	Female	P value
C2-3	56.99±12.22	53.98±10.14	0.40	31.76±3.20	30.60±4.92	0.211
C3-4	59.89±10.12	58.62±9.31	0.68	33.56±4.83	31.94±4.97	0.14
C4-5	57.81±10.20	59.33±14.65	0.70	33.96±4.67	31.74±4.30	0.30
C5-6	56.95±14.39	56.63±10.04	0.93	32.42±5.08	34.40±4.43	0.06
C6-7	64.00±11.66	64.61±14.14	0.88	34.72±5.88	34.07±4.56	0.58

P value: compared with T2 values between male and female for nucleus pulposus (NP) or anulus fibrosus (AF).

### Pfirrmann classification

**Figure 4:**
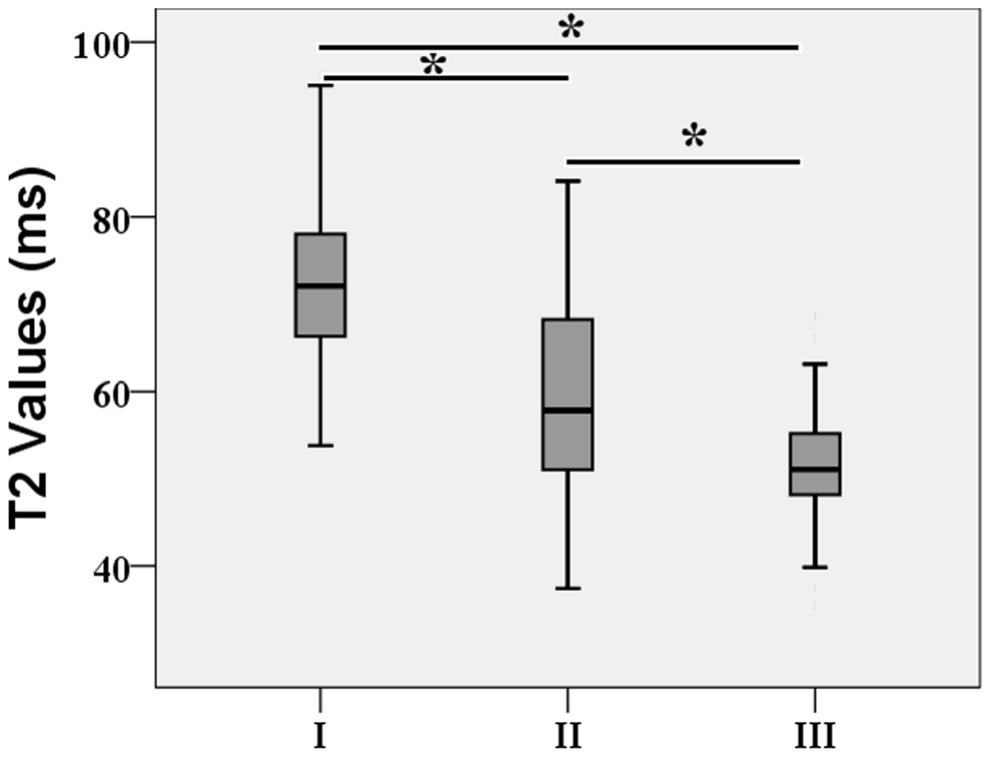
Box plots of T2 value of nucleus pulposus (NP) according to the grade of Pfirrmann. The boxes represent the median and the interquartile range, with the vertical lines showing the range. Three case with Pfirrmann grade IV (T2 value = 36.72) was excluded. *P<0.000.

Table 5 lists the mean T2 relaxation times for different regions for each Pfirrmann grade. Grade I discs had markedly higher NP T2 values than all of the other discs. There was a stepwise decrease in NP T2 values from grade I to grade III, with highly statistically significant differences between each grade (all P<0.000; Table 5 and Fig. 4). Spearman correlation analysis revealed a strong negative association between the Pfirrmann grade and the T2 values of the NP (r = −0.561; P<0.000) but not the T2 values of the AF (r = −0.009, P = 0.854). The total T2 values were 59.03±11.94 ms for the NP, 31.32±4.84 ms for the anterior AF, and 34.46±4.36 ms for the posterior AF for all discs. There was no statistically significant difference in T2 values between the anterior AF and posterior AF. The NP, anterior AF, and posterior AF values did not differ significantly between male and female subjects at the same anatomic level (P>0.05). However, the interquartile range shows the wide heterogeneity distribution of T2 values for the NP, especially for the non-degenerated discs identified based on the Pfirrmann scale. The T2 value ranged from 53.80 to 96.28 ms for the 79 grade I discs, 37.42 to 84.10 ms for the 145 grade II discs, and 35.42 to 68.70 ms for the 123 grade III discs.

**Table 5:** T2 values for discs at different Pfirrmann grades.

Grade		T2 values (Mean±SD, ms)		
	N	AAF	NP	PAF
I	79	32.14±5.15* ^@^	72.25±10.87^@^	34.92±3.90* ^@^
II	145	30.73±4.25* ^@^	59.36±11.00*	34.21±4.82* ^@^
III	123	31.62±5.33* ^@^	51.73±6.33* ^@^	34.54±3.98* ^@^
IV	3	22.41	36.72	23.28
P value	—	>0.05	<0.000	>0.05
Total	350	31.32±4.84	59.03±11.94	34.46±4.36

* Compared with T2 values of nucleus pulposus (NP) of grade I, P<0.05;

@Compared with T2 values of NP of grade II. P value: compared with each anterior anulus fibrosus (AAF), NP and posterior anulus fibrosus (PAF) among grade I, II, and III.

### ROC analysis

**Figure 5:**
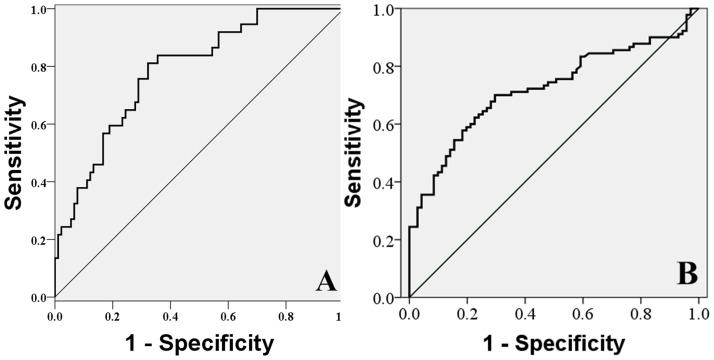
Receiver-operating characteristic (ROC) curve between each grade in the NP and cut-off value. The most accurate point was obtained from the ROC curve as the cut-off value. A. The T2 cut-off value between grades I and II was found to be 62.03 ms, sensitivity 83.80%, specificity 64.40%, and Area Under curve (AUC) 0.785, separately. B. The values between grades II and III were 54.60 ms, 66.80%, 71.18%, and 0.722, separately.

Comparison of the ROC between each grade for the NP yielded the cut-off values shown in Figure 5. The T2 cut-off value between grades I and II was 62.03 ms, which corresponded to sensitivity, specificity, and area under the ROC curve values of 83.80%, 64.40%, and 0.785 separately; the cut-off value between grades II and III was 54.60 ms, with corresponding values of 66.8%, 71.18%, and 0.722, separately (Fig. 4).

## Discussion

IVD degeneration results from multiple environmental factors and the physiological consequences of aging. It is correlated with mechanical factors related to long-term heavy load physical activity, long-term fixed positions [3], and commonly linked to aging [27], [28]. However, early cervical IVD degeneration also can occur. Jiang et al [29] reported that stable and mature IVDs changes begin to develop early degeneration when humans are between 16 and 25 years old. The degenerative process can cause a series of pathological changes and clinical symptoms that correspond to imaging findings, such as disc bulging, protruding, or prolapse. Our previous pilot experiments confirmed that many adolescents have IVD degeneration but are asymptomatic. However, researchers also have suggested that physiological (i.e., non-traumatic) changes in the IVDs likely begin as early as the second decade of life and progress until death [30]. Then previous author [31] found no remarkable changes in the biochemical components between normal and cervical IVD herniation without cord and nerve root compression, which indicates that the early stages of degeneration may be reversible.

The IVD changes, especially PG organization and water concentration, begin during growth and development, well before evidence of disc degeneration appears [32]–[34]. Early signs of disc degeneration are manifested by biochemical changes (e.g., PG loss, dehydration, and collagen degradation) that eventually lead to morphologic degradation in the vertebral bodies, endplates, and facet joints [22]. Although a single initiating cause of degeneration has not been identified, early degenerative changes are known to occur in the NP [34]. Degeneration appears to be initiated by the incapacity of disc cells to maintain a highly hydrated PG-rich matrix in the NP and by loss of the collagen structure that affects the mechanical integrity of the IVD [35], [36]. Thus, IVD degeneration is a complex and multifaceted process, and the associated biochemical and morphologic changes adversely affect the mechanical and functional integrity of the disc.

### Characteristics of cervical IVD T2 values in young adolescents

The strength of this study is the large homogeneous asymptomatic population of young adults analyzed with unconventional *versus* standard MRI techniques. To our knowledge, T2 mapping of cervical IVDs in such population has never been reported previously in the peer-reviewed literature. Early degenerative changes were quantitatively assessed by T2 relaxation time revealing a no statistically significant differences at any anatomic position between sexes. These results suggest that water loss from the NP or AF in cervical IVDs may begin at the same time for both genders. Moreover, IVD changes assessed by T2-WIs MRI Pfirrmann grading (grade III–IV) were observed in 35.99% of the subjects, and the non-degenerated discs (grade I–II) were just in the 64.01%, Although IVD degeneration is known to be common among asymptomatic patients [23], the prevalence of MRI findings among young adults is virtually unknown.

In the present study, the mean T2 values in normal cervical discs was lower than those previously reported in healthy lumbar discs [15], [17], [22], [37]. This difference may due to the different biochemical properties characteristic of cervical and lumbar IVDs. Our T2 values for the cervical IVDs consisted with previous author's report, who [38] investigated the age-related histological and chemical changes that occur in cervical, thoracic, and lumbar AF and NP in human IVDs and found that water content of the AF was lower than that of the NP, with a trend towards higher values in the order cervical < thoracic < lumbar (and the trend was stronger in the NP).

Segmentation motion of the cervical spine is lowest at the C2–3 level and greatest at the C4–5 and C5–6 levels [39]. Degenerative processes are most prevalent in the C5–6 segment, followed by C6–7 and C4–5 [40]. Our results agree with these previous reports (C5–6, grade III, 32 of IVDs) but vary somewhat in terms of C4–5 and C6–7. Morishita et al [39] reported that few of their subjects had C3–4 and C4–5 segments that were grade III, which were not agree with our results due to their relatively small samples choosing and appraising by insensitivity of Pfirrmann grade.

Compared to the IVD of the lumbar spine, cervical rotation and translation movements with resulting torsion and shearing forces to fibers of the AF may explain the more pronounced histological changes in the AF of the cervical spine, which supports the premise that mechanical loading plays a role in IVD degeneration [33]. However, these local changes in the AF are not well detected by the macroscopic classification system [22]. Some studies have shown that microscopic cracks within the AF appear during the second decade of life and that these cracks increase in number and size in the third decade, particularly in the posterior part of the AF [41]. Stelzeneder et al [17] reported that the posterior AF T2 values were higher than the anterior values in the lumbar spine among subjects aged 15 to 64 years old. The same results were reported in some studies [19], [22] and were attributed to physiological variation of the compactness of the AF fiber network between the anterior and posterior regions. In our study, there was little difference between the T2 values of the anterior and posterior AF. Possible explanations for the similarities in values observed in our study are that physiological changes in the AF likely occurred at the same time in the young adolescents and that the mechanical properties of the biophysical structures of the anterior AF and posterior AF differed between the lumbar and cervical spine [38].

Because there were no cases with grades indicative of serious damage in our sample of young adolescents, we found no correlation between the T2 values of the AF and Pfirrmann grades. Only 3 of 70 subjects in our study were assigned Pfirrmann grade IV, and they had significantly lower T2 values for the NP, anterior AF and posterior AF than the other grades (22.41 ms, 36.72 ms, and 23.28 ms, separately). Previous studies have demonstrated that T2 values in the NP and AF decrease in proportion to disc degeneration, and, ultimately, differences in T2 signal intensity between these two structures approach zero [37], [42]. This premise is consistent with our results, especially for grades III and IV. Thus, comparison of T2 values between the NP and AF may also aid in the evaluation of disc degeneration.

Comparison of T2 values with morphological grading revealed a stepwise decrease in NP T2 values with increasing Pfirrmann grade and a strong negative rank correlation. This pattern might be due to decreasing water content and structural disorganization with increasing grade of disc degeneration [43]. T2 relaxation time is sensitive to water content and the arrangement of the collagen network structure [44]. However, molecular factors also take influence on relaxation times. One important factor could be the water concentration itself. If water is pressed out of the vertebral disc matrix, the relative concentration of water decreases and the effects of the macromolecules may rise, resulting in shortened relaxation times [45], [46]. T2 decrease with the decrease of water and PG content associated with disc degeneration [47] and then a higher T2 for the NP has been shown in healthy IVD. However, non-degenerated discs (Pfirrmann grades I and II) showed a wide range of T2 relaxation times in our results (Fig. 4), which also was observed in previous studies [24], [28], [37] in lumbar IVDs. Sowa et al [48] examined the changes observed during normal aging of the IVDs in an animal model and found modest age-related MRI changes assessed using T2-weighted images. In contrast, dramatic histological changes were found for the same discs, which suggest that conventional MRI techniques are relatively insensitive measures of IVD degeneration.

T2 values measured in this study was higher at caudal levels in both the NP (C4–5 to C6–7) and the AF (C2–3 to C6–7), with an increasing trend of T2 values proceeding cranially up the spinal column. This new finding is contradicted with observations in lumbar spine by others with different evaluation techniques [25], [49], [50]. We interpret this observation to support the hypothesis that higher T2 values reflect more water and PG to resist more mechanical loads (torsion and shearing forces) on lower cervical discs (compared with biomechanical stress on more cephalic discs). Further study (eg, in human cadaveric samples), however, will be necessary to prove this finding.

ROC analysis between each grade of the NP yielded cut-off values for quantification of disc degeneration based on T2 signal intensity. The cut-off points obtained from the ROC curve were determined based on area under the curve (AUC) values. AUC values of 0.9–1.0 denote high accuracy, 0.9–0.7 indicate moderate accuracy, and 0.5–0.7 represent low accuracy. The AUC values in our study were all within the moderate accuracy range, indicating a moderate level of reliability (Fig 5). These data indicate that this T2 value-based grade scale for the NP is useful as it has a moderate degree of objectivity. In addition, as in previous results [17]–[19], good to excellent intra- and interobserver agreements were observed in this study. Thus, sagittal T2 mapping has the potential to replace or supplement conventional classification systems in grading early degenerative IVDs.

### Factors affecting T2 values

T2 values can be affected by many factors, including gender, age, measure method and area, and testing time. Several studies have shown that age-related IVD degeneration can be detected based on T2 changes and that age-related reduction in T2 is associated with decreased glycosaminoglycan and water content [16], [21] or sex variance [30], [50]. The water content changes in IVDs occur due to posture and activity. Fluid is expressed from the disc when it is compressed and is reimbibed when the load is removed during the ongoing cycle of high loads during daily activities and low loads at night during rest [51]. Karakida et al [45] examined asymptomatic volunteers and documented diurnal T2 value changes in both normal and degenerated discs. Ludescher et al [24] also reported diurnal T2 value changes from morning to evening and confirmed that the T2 values of both the NP and AF changed in opposite directions. To minimize the influence of diurnal variation on T2 values in IVDs, we performed MRI at a fixed time in the afternoon for all volunteers. T2 relaxation times also change during the period of human growth and maturation [15]. Krueger et al [15] measured mean T2 relaxation times for volunteers less than 10 years old and for 19–20 year olds and showed that T2 values increase between the first and second decades of life.

Five or seven equally sized rectangular ROIs on the two adjacent central slices were manually drawn on the lumbar sagittal images following previously reported procedures [17], [19], [28], [52]. However, inappropriate choice of ROIs (due to differing anatomical characteristic between cervical and lumbar regions) can create huge partial volume effects. To evaluate ROIs in a reproducible way, we adopted trisection methods [18], [22], [26], [50] in order to decrease selection bias. Nagashima et al [26] proposed that the ROIs should be determined so as to allow discrimination of subtle differences in SI between the IVDs. In their method, the shape of the ROIs was determined such that the quadrangle formed by the anterior and posterior edges of the upper and lower end plates in contact with the IVD to be measured was defined as the intervertebral area. A shape similar to the intervertebral area but with one-quarter the area was drawn in lumbar IVDs. The geometrical center of the shape was matched to the center of intensity and was confirmed to have satisfactory validity and reproducibility. Therefore, in our study an ellipse was drawn according on the cervical anatomical characteristics. For discs with Pfirrmann grades of III and IV, for which the boundary between the NP and the inner AF was not clearly distinguishable, an irregular ROIs was delineated by the operator according to the expected location of the inner portion [47], and these ROIs on T2WIs were copied to the T2 maps at same anatomical level.

### Limitations

This study had several limitations. First, the manual delineation of the ROIs to cover the inner portion of the IVDs could have introduced subjectivity and bias, especially when the central region was poorly differentiated from the outer AF region in discs with Pfirrmann grades of III and IV, IV and V; however, only three discs in our study fell into this category. Second, we did not compare results of advanced imaging techniques such as T1p, T2* mapping, or ADC [37], [50], [53] with results of T2 mapping, but many previous studies have confirmed the usefulness of T2 mapping alone to diagnose in IVD degeneration [14], [17], [28], [47]. Third, no histological analysis of IVDs was conducted. Further study (eg, in human cadaveric samples) will be necessary to validate this finding.

### Conclusions

In summary, segmental quantitative T2 evaluation seems to be an effective method for quantitatively characterizing different degrees of disc degeneration. This study provides detailed data about T2 relaxation times at 3.0-T in discs at different stages of degeneration that can be used to diagnose early cervical IVD degeneration in healthy adolescents. The wide distribution of T2 values in healthy IVDs highlights the low sensitivity of Pfirrmann grading to detect early changes in IVDs. In contrast, quantitative T2 mapping of the cervical spine is a promising method, particularly for longitudinal follow-up. The use of T2 map images in clinical protocols may prove to be useful in the development of treatments (e.g., cell therapy) for early cervical IVD degeneration.
